# Tyrosine Kinase Inhibitors and Pregnancy

**DOI:** 10.4084/MJHID.2014.028

**Published:** 2014-04-07

**Authors:** Elisabetta Abruzzese, Malgorzata Monika Trawinska, Alessio Pio Perrotti, Paolo De Fabritiis

**Affiliations:** Hematology, S. Eugenio Hospital, Tor Vergata University

## Abstract

The management of patients with chronic myeloid leukemia (CML) during pregnancy has become recently a matter of continuous debate. The introduction of the Tyrosine Kinase Inhibitors (TKIs) in clinical practice has dramatically changed the prognosis of CML patients; in fact, patients diagnosed in chronic phase can reasonably expect many years of excellent disease control and good quality of life, as well as a normal life expectancy, including the necessity to address issues relating to fertility and pregnancy. Physicians are frequently being asked for advice regarding the need for, and/or the appropriateness of, stopping treatment in order to conceive. In this report, we will review the data published in terms of fertility, conception, pregnancy, pregnancy outcome and illness control for TKI treated CML patients, as well as how to manage a planned and/or unplanned pregnancy.

## Introduction

The hybrid BCR-ABL gene and its tyrosine kinase constitutionally active recombinant fusion protein (p210 BCR-ABL) deriving from the reciprocal translocation between chromosomes 9 and 22 is associated with the clinical development of chronic myeloid leukemia (CML).^1^–^2^ This fusion results in the expression of two forms of protein-tyrosine kinases: p190 (BCR-ABL) and p210 (BCR-ABL) with subsequent dysregulation of intracellular signaling that drive cells to enhanced proliferative capability and resistance to apoptosis.

The presence of this well defined pathogenetic defect at the molecular level led to the development of Imatinib, a tyrosin kinase inhibitor able to block the BCR-ABL aberrant molecule, thus shutting down the leukemia phenotype.^3^–^4^

Imatinib (Glivec, Novartis), is the first of a series of tyrosin kinase inhibitors (TKIs), a group of drugs used to manage patients with chronic myeloid leukemia (CML) through the competitive ATP inhibition at the catalytic binding site of the bcr-abl protein.^5^ The second and third generation TKIs include Nilotinib (Tasigna, Novartis), Dasatinib (Sprycel, Bristol Myers Squibb), Bosutinib (Bosulif, Pfizer), and the recently approved Ponatinib (Iclusig, Ariad Pharma).

The introduction of TKIs in clinical practice has dramatically changed the prognosis of CML patients. Data derived from first line therapy (IRIS Study) at 7 years follow up, confirmed one year later, reports cumulative best rates of complete cytogenetic remission (CCR) of 82%, and an estimated overall survival of 89%.^6^–^8^

Patients diagnosed in chronic phase can reasonably expect many years of excellent disease control and good quality of life (QoL); furthermore, patients in an optimal response can reach a life expectancy similar to the non-leukemic, same age, population.^9^

**Figure 1:**
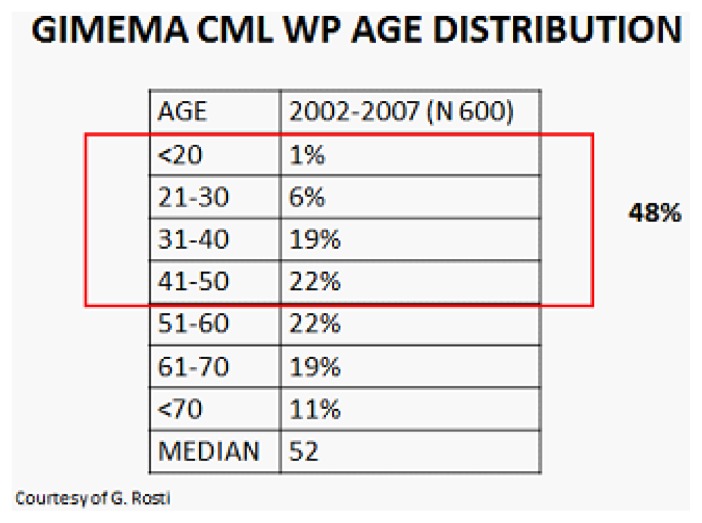


Even if a higher median age at diagnosis (55–60 y.o.) were reported, the GIMEMA registry of CML has reported that approximately 50% of patients at diagnosis are in reproductive age (Figure 1). This has addressed issues relating fertility and pregnancy and physicians are frequently asked for advice regarding the need and/or the appropriateness of stopping treatment in order to conceive.

## TKIs in Animal Model

### Imatinib

Studies on either males or females rats and mice have shown that Imatinib administered to fertile animals has a teratogenic but not gonadotoxic activity.

However, when male rats were given Imatinib at dosages between 20 and 60mg/kg (corresponding to the human dose of 200–600mg/d) lower testicular weight and reduction of sperm mobility were observed at higher dosage. If similar dosages were given to immature rats, interference with the normal process of testis maturation was noticed, while, at sexual maturity, a normal number of sperm counts, motility, maturation and development and higher levels of FSH and LH were registered.^10^ Effects of Imatnib on ovary were unremarkable, so that the fertility of male and female rats was not affected.

The effects on gestation differed by the dosage utilized: teratogenic effects of skull and bone formation (exencephaly, encephalocele, absent or reduced frontal bones and absent parietal bones) were seen when Imatinib was administered during organogenesis at 100mg/kg (corresponding to 1000 mg in humans), while, at higher dosages, total fetal loss was seen in all animals. Fetal loss was not observed at dosages <=30 mg (Novartis: Imatinib investigator brochure).

### Nilotinib

As reported with Imatinib, genotoxicity studies in bacterial in vitro and in vivo mammalian systems did not reveal evidences for a mutagenic potential of Nilotinib.

In pharmacokinetic distribution studies in rats at dosages up to 180mg/kg per day, Nilotinib showed minimal brain and testis penetration. A significant decrease in total epididymal weight was observed at the maximum dose level, while all other male reproductive parameters, including sperm count and sperm motility, were unaffected.

Reproductive and developmental studies have been completed in rats and rabbit. No effects on fertility were noticed in males or females rats, while at doses >20mg/kg/d the embryos died. Compared to Imatinib, no evidence of teratogenicity in rabbits or rats was seen, while the drug was embryo- and fetotoxic in rats and rabbits at dosage producing maternal toxicity. The oral administration of Nilotinib in female rats from d6 gestation to d21 post-partum resulted in only maternal effects, as observed after longer gestational period, reduced food consumption and lower body weight gain at 60mg/kg. The maternal dose of 60mg was also associated with decreased pup body weight and changes in some minor physical, developmental parameters (earlier tooth eruptions and eye opening).

Following a single dose of 20 mg/kg oral dose of C14 Nilotinib in pregnant rats, the higher tissue concentration compared to blood were observed in the maternal liver, kidney, uterus heart and amnion. In the fetus, the tissue concentration was lower than that observed in the maternal organs, except for the liver, which had 1.6 fold higher levels. After oral daily dosing between 30 and 100 mg/kg, Nilotinib concentration was below the limit of quantification in rabbit’s fetuses, while in the group treated with 300mg/kg; fetus concentration was only 8% of the maternal serum, indicating a low absorption of Nilotinib in the fetus. The transfer of drug and metabolites to milk was observed following an oral dose of C14 Nilotinb to lactating rats. It is estimated that, in 1L of human milk, the infant can be exposed to 0.26% of a 400mg adult dose. (Novartis: Nilotinib investigator brochure)

### Dasatinib

Dasatinib did not appear to affect fertility in male rats at dosage <10 mg/kg/day and was not toxic to the offspring at these doses, while effects included reduced size and secretion of seminal vesicles and testis and an immature prostate.

In female rats, Dasatinib was observed to be teratogenic, extensively distributed in maternal tissues, and secreted into milk.^11^

At plasma concentrations below those observed in humans receiving therapeutic dosage of Dasatinib, embryo-fetal toxicities were observed in pregnant rabbit and rats. Fetal deaths were observed in rats. In both rats and rabbits, the lowest doses of Dasatinib tested resulted in embryo-fetal toxicities. These doses produced maternal AUCs 0.3 fold the human AUC in females at dose of 70 mg twice daily, and 0.1 fold the human AUC in rats and rabbits. Embryo-fetal toxicities included skeletal malformations at multiple sites (scapula and all long bones, including ribs), reduced ossification, generalized oedema and microhepatia. (Dasatinib Investigator Brochure; Sprycel® (Dasatinib) tablets, Summary of Product Characteristics)

It is not known whether Dasatinib is excreted in human milk.

### Other TKIs

Little is known concerning the recently approved Bosutinib (Bosulif, Pfizer) and Ponatinib (Iclusig, Aria Pharma), although for both of them preclinical studies of mutagenesis seem to be negative, and teratogenity is similar to the other described TKIs with effects on osteogenesis and vasculogenesis.

## TKIs in Pregnancies and Conception

### Imatinib in man

Since the first reports of unplanned conception in male taking Imatinib at standard and higher dosages, no increased risk of congenital malformations or increased abortion have been reported.^12^–^14^

In a series of female and male patients treated with Imatinib, Ault et al^15^ described 8 male patients who conceived, with an exposure of 18 months (range 4–48 months) to Imatinib. Taking into consideration the 72 days for male gonads to come to maturation, the majority of those patients have been fully exposed to the drug at conception. In those 8 patients, conception resulted in the birth of 8 babies (1 spontaneous abortion and one twin pregnancy). One of those babies was born with a mild malrotation of the small intestine, surgically fixed. No problems were observed after the birth in terms of growth and development in 38 months follow up.

More than 150 cases have been described so far and except for the malrotation of the intestine reported earlier and one stillbirth with malformations in the previous series, the other pregnancies/deliveries resulted uneventfully.

### Imatinib in women

Completely different is the outcome when the woman is taking Imatinib, and the embryo-fetus is exposed to the drug.

Different reports have been published in the past years reporting patients treated with Imatinib who conceived and or became pregnant during therapy. In the series of Ault et al., together with the 8 male patients, 10 female were presented (1 in CCR, 1 in accelerated phase and 8 in an advanced phase of the disease) who received Imatinib at standard dose (9 patients) or 800mg (1 patient) until pregnancy was identified, with exposure between 4 and 9 weeks. Two patients had a spontaneous abortion after discontinuation of Imatinib, while one patient had a therapeutic abortion. The remaining seven pregnancies were carried to term resulting in the birth of 8 babies (twin girls). One baby had a hypospadias that was surgically corrected, while the remaining 7 were healthy and presented with a normal growth and development after 53 month follow up.

The first systematic review of pregnancies was reported by Pye et al,^16^ who collected information from different Institutions, describing 180 women exposed to Imatinib during pregnancy. Of these pregnancies, outcome data are available for only 125 (69%). Women were exposed to Imatinib during all pregnancy (38 patients), during 1^st^ trimester (103 patients), during both 1^st^ and 2^nd^ trimester (4 patients) or after the 1^st^ trimester (4 patients). Of those with known outcomes, 50% delivered normal infants and 28% underwent elective terminations, 3 following the identification of abnormalities. Of 12 infants with abnormalities identified in carried pregnancies, 8 were live births, 1 stillbirth, and 3 terminations. A total of 10 of the 12 infants with abnormalities have been exposed to Imatinib during the first trimester (information unavailable for the remaining 2 infants). Because of both tyrosine kinases and bcr-abl inhibition by Imatinib, it is conceivable that the congenital abnormalities result from inhibition of members of this extensive family. Furthermore, the abnormalities evidenced were similar to those observed in preclinical studies (exencephaly, encephalopathies and abnormalities of the skull bones observed in the rodent studies).

Recently, more attention is given to the possibility of a planned/unplanned pregnancy in CML patients of both sexes. The majority of the events described, including 2 cases from our institution (unpublished data), are carefully followed, pregnancies are possibly planned, and Imatinib interrupted early or right before conception, so all of the pregnancies resulted in normal babies. The behavior of both physicians and patients may be different in other Countries where discontinuation of the drug is not systematic, and most of the pregnancies were not even reported to the hematologist when discovered.^18^–^19^ Of the 28 pregnancies from 21 female patients in remission reported by the Pakistan group, 27 assumed Imatinib. Of the 27 exposed pregnancies, 6 were exposed during 1^st^ trimester (wks 6–12), 12 during 1^st^ and 3^rd^,^14^–^31^ and 9 throughout the pregnancy until delivery. One stillbirth presented with congenital malformations and one baby died without malformations after one week after an apparently uneventful pregnancy. In this series, the authors reported as adverse event 2 babies born prematurely with low birth weight. Finally, three spontaneous abortion and 3 elective abortions were recorded.

Table 1 summarizes 167 of a total 210 Imatinib female pregnancies published and/or observed in our Institution with sufficient data and follow up. Within those 167 pregnancies, 128 were uneventful (77%), while 24 ended in spontaneous abortion (14%), a percentage slightly higher compared to the normal population (10–12%).^17^ Fifteen/167 (9%) presented with abnormalities, including one referred to a concomitant drug (warfarin syndrome). All patients in this group were exposed to Imatinib during organogenesis (>5wk gestation).

Finally, although most pregnancies exposed to Imatinib may have a successful outcome, a significant proportion of drug-related serious fetal malformations and a slightly higher risk of spontaneous abortion remain at risk. For this reason, pregnancy should not be avoided, but planned.

### Nilotinib in men

Little is reported regarding conception during Nilotinib treatment. One case from our Institute (unpublished data) regards a 33 years male patient with CML enrolled in the GIMEMA NILIM trial (alternated Nilotinib/Imatinib), who wanted to conceive his 2nd child. He discussed the opportunity to delay therapy but, taking into consideration the 72 days necessary to complete gonads maturation process in male, we suggested conceiving in the first two months on Nilotinib therapy. He conceived after 40 days and had a healthy boy born at term. He is now 5 years old, regularly growing and healthy.

The investigator brochure, ed.8 (June 2012) refers to a total of 36 cases of drug exposure via the father. One of these cases presented with fetal abnormalities ended in a therapeutic abortion.

### Nilotinib in woman

As for men, very little is reported for women getting pregnant while exposed to Nilotinib. Two cases were published and indexed, and one more case was reported by an Italian institution. The firstly published case regards a 30-year-old woman with chronic myeloid leukemia who became pregnant twice successfully. Philadelphia-positive CML in its chronic phase was diagnosed at 16 weeks of her first gestation and received no treatment throughout her pregnancy. At 38 weeks of gestation, a normal infant was delivered by cesarean section. Two years later, while, in major molecular response (MMR) on Nilotinib 200 mg bid, she became pregnant again. The unplanned pregnancy was identified during her first trimester of gestation after the patient had experienced 7.4 weeks of amenorrhea. The patient was informed of the potential fetal toxicities of therapy, but decided to carry on her pregnancy. Nilotinib was stopped, and no further treatment was given until delivery. A follow-up with ultrasound scans during the course of the pregnancy was unremarkable. At gestational week 33, she delivered via cesarean section a healthy male baby weighing 3.2 kg. He was breast-fed for 2 months and at 5 months post-partum, the child was healthy and normally developing..^20^

The second case was reported by the French intergroup of CML.^21^ It regarded a 38 years female who got her 5^th^ pregnancy while on Nilotinib. Treatment was stopped when pregnancy was discovered, and replaced by Interpheron-a. Three months ultrasound showed a big omphalocele ending into the pregnancy interruption.

The other case, unpublished, was reported in Italy: a 41 years female resistant to Imatinib, switched to Nilotinib 400 bid, achieving MMR after 3 months, and complete molecular remission (CMR) with bcr-abl transcript undetectable at 9 months. After 11 months she became pregnant. Fetus was exposed for 5 weeks before stopping therapy. The pregnancy was unremarkable. She lost CMR, but PCR stayed <0.19% IS until delivering a healthy baby girl.

In the Nilotinib investigator’s brochure, 45 cases have been reported of drug exposure during pregnancy. There was only one case with fetal abnormalities (probably the same already described by the French group). In addition, there was one female exposed pregnant of twins with one twin experiencing congenital transposition of great vessels resulting in death, and the other twin experiencing a non-serious heart murmur.

### Dasatinib in men

Cortes et al evaluated the effect of Dasatinib on pregnant partners of 9 male patients who conceived children while receiving Dasatinib (22). Normal newborns were reported in 7 cases, with the outcome of the other cases unknown. All male patients remained on treatment during and after the pregnancies. In 1 case, the mother experienced pre-eclampsia but delivered a healthy newborn at 37 weeks, without birth defects or neonatal complications.

### Dasatinib in woman

Published information regarding the use of Dasatinib in pregnancy is limited to 17 cases describing the outcome of female patients with CML, who became pregnant while receiving Dasatinib. In all but 1 case, Dasatinib was stopped upon confirmation of pregnancy in patients electing to give birth. Data from 16 patients with CML enrolled in phase 1 to 3 Dasatinib clinical trials and 6 voluntarily submitted reports were used to complete the study described by Cortes et al..^22^ A total of 13 pregnant female patients were identified: of these, 1 patient gave birth to a normal newborn, 1 to a premature newborn, 4 had induced abortions, 2 had spontaneous abortions and 5 were pregnant at last reported follow-up. The normal infant was born to a 36-year-old female patient with CML in chronic phase (CP). Pregnancy was identified after 7 weeks of gestation when the patient was under treatment with 70 mg of Dasatinib twice daily. The premature infant was born to a 29-year-old female patient with CML in accelerated phase treated with Dasatinib 70 mg twice daily; he was delivered by caesarian section after 7 months’ gestation, was “small for dates” but had no defects.

Kroll et al^23^ reported the use of Dasatinib in a 23-year-old female patient with CP-CML, who became pregnant while undergoing treatment. When pregnancy was identified after approximately 5 weeks of gestation, she was being treated with Dasatinib 70 mg once daily and was in CCR. Treatment was discontinued immediately. While the patient was closely monitored and off therapy, she developed leukocytosis and a mild thrombocytosis. Subsequently, she was given hydroxyurea, but developed progressive leukocytosis and increased levels of lactic dehydrogenase levels. Cytarabine was administered to reduce the white blood cells (WBC) count to normal within several days. To avoid further fetal exposure to chemotherapy and resume more definitive therapy for the mother’s CML, doctors induced labor at 35 weeks. The patient delivered a healthy female baby without any developmental delay or structural and functional anomalies. Three days post delivery, Dasatinib 70 mg daily was restarted concurrently with a 2-week hydroxyurea tapering. She reached CCR in the peripheral blood 5 months after re-initiating Dasatinib.

Conchon et al^24^ reported the use of Dasatinib in a 25-year-old female patient with CP-CML, who became pregnant while undergoing treatment with 70 mg twice daily. Pregnancy was identified during the first trimester, while she was in hematologic remission and Dasatinib was discontinued immediately. Hematologic relapse occurred, and the patient was, therefore, treated with INF-α (although complete hematologic response was not achieved). The patient delivered a male baby at 33 weeks with no sequelae or malformations. A few days following delivery, the patient was treated with hydroxyurea for 4 months, and then restarted TKI.

Berveiller et al^25^ reported a 23-year-old woman with CML, who became pregnant after the switch to Dasatinib following Imatinib failure. When pregnancy was identified at 9 weeks of gestation, she was treated with Dasatinib 100 mg once daily for 4 weeks achieving complete hematologic response. Dasatinib was not discontinued due to the high-risk characteristics of the patient, and an obstetric ultrasonography at 16 weeks of gestation revealed a fetal hydrops with subcutaneous edema, pleural effusion, and ascites. Pregnancy was terminated due to the poor perinatal prognosis after 17 weeks of gestation. The patient delivered a eutrophic male fetus with no organ malformations except for microretrognathia and hypertelorism. Fetal karyotype was also normal. Fetopathologic examination revealed a subcutaneous edema in the nuchal and dorsal regions. Transplacental transfer of Dasatinib was observed with drug concentrations of 4 ng/mL in maternal plasma, 3 ng/mL in fetal plasma, and 2 ng/mL in amniotic fluid.

Bayraktar et al^26^ reported a normal pregnancy outcome of a 25-year-old patient with CML, who was treated with Dasatinib 100 mg once daily for the first 6 weeks of gestation. Once the pregnancy was confirmed, Dasatinib was stopped, and the patient was managed conservatively with close observation of her disease. During her pregnancy, hematologic relapse occurred with mild leukocytosis and thrombocytosis that did not require treatment. The infant was delivered at 37 weeks without any documented birth defects. The patient was restarted on Dasatinib 100 mg daily shortly after her delivery and did not breastfeed. At 2 years’ follow-up post delivery, the patient had molecular remission and her daughter met all developmental milestones.

### Other TKIs

As far as we know, no conception/pregnancies were described while taking Bosutinib or Ponatinib. In our Institution, we followed a male patient diagnosed with CML and enrolled in a Bosutinib trial that cryopreserved his sperm before starting therapy and conceived with an intra-uterine insemination (IUI) a healthy baby girl who is now 3 years old and normally developing.

## Discussion

Imatinib and the subsequent second and third generation TKIs has represented and represents, a major advance leading to a successful targeted therapy with substantial improvement of survival and quality of life in CML patients.

Considering the significant proportion of female/male patients diagnosed with CML in reproductive age, and the substantial normal lifespan of those patients when treated and responding, it became mandatory to address issues relating to fertility and pregnancy. The management of fertility begins at diagnosis. In fact, the patient in reproductive age should be informed about the risk of unplanned pregnancies in terms of fetal problems and/or the risk of uncontrolled disease in the case of stopping therapy, but also on the possibility that a controlled pregnancy can be carried out when the treatment has being started, and the response is optimal.

There are no consensus/guidelines regarding the best behaviour in case of pregnancy.

While it seems that there are no problems in terms of fertility, conception and delivery of female partners of male patients (even if for newer TKIs not enough data are available), female patients should not be exposed to TKIs during pregnancy.

Based on the published data, 10–20% of maternal exposure during the 1^st^ trimester to TKIs ends in fetal problems or spontaneous abortion. The problems consist mainly in skeletal malformations, soft tissue abnormalities (especially involving the vessels and organs formation) and small for date babies, and are similar to the one described in animal studies.^27^

An independent algorithm by Kumar et al. for the management of CML during pregnancy recommended discontinuation of TKIs. If patients are in the first or second trimester, interferon alpha (IFN-α) can be given; leukapheresis can regulate white blood cell (WBC) counts as required and hydroxyurea considered for patients not responding to IFN-α. Patients in their third trimester and not responding to IFN-α or hydroxyurea can be treated with Imatinib, and if still not responding, with second generation TKIs.^28^

Shapira et al suggested an algorithm in which pregnancies discovered at diagnosis should be considered for Imatinib treatment only if unresponsive to IFN-α and, possibly, after 1^st^ trimester.^29^

Imatinib is a compound that highly binds to plasma proteins and has a high molecular weight that should limit placental transfer. Two studies addressed the possibility to administer Imatinib later in the course of pregnancy, evaluating the concentration of this drug and its active metabolites in the different maternal/fetus compartments. In two cases presented by Russell at al, Imatinib appears to cross the human placenta poorly. In two pregnancies taking Imatinib during the 3^rd^ trimester, concentration of drug and his metabolite CGP74588 was measured at delivery in maternal blood, placenta, and cord blood. Little or no drug was found in the cord blood, while it was present at high concentration in maternal blood and placental tissue, confirming this hypothesis.^30^ In a different pregnancy with exposure from 21^st^ to 39^th^ week of gestation, Imatinib was present at 338ng/mL in the cord blood and 478 ng/mL in the peripheral blood infant (1/3 range) vs 1562 ng/mL in maternal blood.^31^

In contrast, an high concentration of drug was found in breast milk in both studies, as confirmed in other works and described in animal models.^32^

These reports suggest that in a male patient taking Imatinib or Nilotinib, no particular risks of fertility; conception or pregnancy have been evidenced. Caution should be used when patient is taking Dasatinib, due to the very few data available. No reports are available for patients taking Bosutinib or Ponatinib. For those patients, the possibility to cryopreserve sperm before starting therapy should be discussed.

In a female patient, in reproductive age, effective contraception should be suggested at diagnosis. A pregnancy should be planned only after the milestone of a stable MMR (or better, e.g. >MR4.5) reached more than 18–24 months earlier. Ob-gyn visit for preconception tests (including in some cases male sperm evaluation), ultrasound and planned conception is highly recommended.

Therapy should be stopped immediately before or soon after conception. All drugs must be avoided during the organogenesis (post menstrual days 31–71, weeks 5–13), Q-PCR must be monitored each month/2 months to follow the transcript. Therapy should be considered only if a cytogenetic or hematologic relapse occurs.^33^ Each single patient should be individually evaluated taking into account the rapidity of the relapse, the clinical CML history, and most of all, the pregnancy status (weeks of gestation).

Interferon can be considered safe throughout pregnancy^34^ and hydroxyurea can be used to control leucocytosis after organogenesis.^35^ When necessary, TKIs therapy with Imatinib or Nilotinib could be considered after placenta has been formed and organogenesis completed,^36^ although high Nilotinib concentration has been found in fetal liver, in animal models. Dasatinib, on the contrary, seems to pass the barrier and should be avoided.

All patients can breast feed the first 2–5 days postpartum to give the baby the colostrum. Newborns have very immature digestive systems, and colostrum delivers its nutrients in a very concentrated low-volume form. It has a mild laxative effect, encouraging the passing of the baby’s first stool; that helps to clear excess bilirubin, contains immune cells and many antibodies, immune substances and a series of cytokines and growth factor.^37^ Considering the few days of delay to resume treatment, it could be important for the newborn to access this.

After delivery and providing a good molecular transcript, therapy can be postponed to consent full breast feeding, according to the haematologist judgement.

All cases with good control of the illness at conception and a good response to a specific TKI stopped during pregnancy and resumed after delivery, have re-reached MMR within 3–6 months, confirming the possibility of a safe therapy manipulation during pregnancy.^38^–^40^

In conclusion, we can resume all those informations updating a table earlier presented by Apperly (Table 2). It is important to take into consideration that each case should be considered as a single case in which many factors may play a crucial role: the biology of the illness, the response to treatment, the outcome during the pregnancy, and the willing of the patient should be clearly considered and discussed.^41^–^42^

In our Institution, we have a team composed by haematologists, urologists, ob-gyn and neonatologists, that work together during the planning, the conception, the pregnancy, the delivery and the immediate post-delivery, to assure the best care to mother and child. We have followed 3 CML female conception/pregnancies and 6 male conceptions, all with normal children (1 female patient pregnancy is ongoing) plus many pregnancies of patients affected by lymphomas or acute leukemia. The preservation of fertility is part of our routine when a female/male patient is diagnosed as having a hematologic problem needing therapy.

Through the GIMEMA CML working party, there is an ongoing observational retrospective and prospective multicenter study to register all female pregnancies/male conception in TKIs era and a similar registry is in preparation through the European Leukemia Network of CML Working Party, coordinated by Italy and Russia. We hope that an extensive report of such events will help in managing the possibility to conceive in CML patients in order to give our patients not only a normal lifespan, but also a normal life.

## Figures and Tables

**Table 1:** Outcome in 265 described pregnancies in CML Imatinib-treated patients.

Pregnancy outcome	Total number N=265	(%) with known outcome N=210	Real % (excluding elective termination with no known problems)
Normal Live Infant	128	60% **(210 pregnancies)**	77% **(167 pregnancies)**
Elective Termination	43	20%	excluded
Fetal Abnormality	15	7%	9%
Spontaneous Abortion	24	11%	14%
Unknown	55	0	0

**Table 2:** Management of a female patient with CML planning a pregnancy

Pre-conception	- >18–24 Months stable >Major Molecular Remission - Counselling with Obgyn to check on: fertility routine preconceptional tests monitoring ovulation
TKI interruption	- Interruption of TKI can be done 7–10 days after ovulation (before implant) - Absolutely no TKI therapy between 5–13 weeks, or 31–71 days after last menstrual cycle (organogenesis) - If available, get in touch with the team that will follow the pregnancy-delivery (haematologist, obgyn, neonatologist)
Disease monitoring	Blood counts according to pregnancy follow up Q-PCR every month if no 4.5 CMR* Q-PCR every 2 months if CMR **In case of loss response always consider risk mother/baby** Consider treatment if loss of MMR/CCR Restart treatment if loss of hematologic response
Post delivery	Breast feed the first 2–5 days to give the child colostrum If in MMR/CCR consider continuing breast feeding depending on PCR results Restart treatment with same TKI used before.

* 4.5 CMR corresponds to a BCR-ABL transcript ≤ 0.0032% copies
